# Cerebrospinal Fluid Neopterin as Marker of the Meningo-Encephalitic Stage of *Trypanosoma brucei gambiense* Sleeping Sickness

**DOI:** 10.1371/journal.pone.0040909

**Published:** 2012-07-18

**Authors:** Natalia Tiberti, Alexandre Hainard, Veerle Lejon, Bertrand Courtioux, Enock Matovu, John Charles Enyaru, Xavier Robin, Natacha Turck, Krister Kristensson, Dieudonné Mumba Ngoyi, Gedeão M. L. Vatunga, Sanjeev Krishna, Philippe Büscher, Sylvie Bisser, Joseph Mathu Ndung’u, Jean-Charles Sanchez

**Affiliations:** 1 Biomedical Proteomics Research Group, Department of Human Protein Sciences, University of Geneva, Geneva, Switzerland; 2 Department of Biomedical Sciences, Institute of Tropical Medicine, Antwerp, Belgium; 3 INSERM UMR1094, Tropical Neuroepidemiology, Limoges, France; 4 Department of Veterinary Parasitology and Microbiology, School of Veterinary Medicine, Makerere University, Kampala, Uganda; 5 Department of Biochemistry, College of Natural Sciences, Makerere University, Kampala, Uganda; 6 Department of Neuroscience, Karolinska Institutet, Stockholm, Sweden; 7 Department of Parasitology, Institut National de Recherche Biomédicale, Kinshasa, D. R. Congo; 8 Instituto de Combate e Controlo das Tripanossomíases, Luanda, Angola; 9 Division of Cellular and Molecular Medicine, Centre for Infection, St. George’s, University of London, London, Great Britain; 10 Foundation for Innovative New Diagnostics (FIND), Geneva, Switzerland; 11 Institute of Neuroepidemiology and Tropical Neurology, School of Medicine, CNRS FR 3503 GEIST, University of Limoges, Limoges, France; Albert Einstein College of Medicine, United States of America

## Abstract

**Background:**

Sleeping sickness, or human African trypanosomiasis (HAT), is a protozoan disease that affects rural communities in sub-Saharan Africa. Determination of the disease stage, essential for correct treatment, represents a key issue in the management of patients. In the present study we evaluated the potential of CXCL10, CXCL13, ICAM-1, VCAM-1, MMP-9, B2MG, neopterin and IgM to complement current methods for staging *Trypanosoma brucei gambiense* patients.

**Methods and Findings:**

Five hundred and twelve *T. b. gambiense* HAT patients originated from Angola, Chad and the Democratic Republic of the Congo (D.R.C.). Their classification as stage 2 (S2) was based on the number of white blood cells (WBC) (>5/µL) or presence of parasites in the cerebrospinal fluid (CSF). The CSF concentration of the eight markers was first measured on a training cohort encompassing 100 patients (44 S1 and 56 S2). IgM and neopterin were the best in discriminating between the two stages of disease with 86.4% and 84.1% specificity respectively, at 100% sensitivity. When a validation cohort (412 patients) was tested, neopterin (14.3 nmol/L) correctly classified 88% of S1 and S2 patients, confirming its high staging power. On this second cohort, neopterin also predicted both the presence of parasites, and of neurological signs, with the same ability as IgM and WBC, the current reference for staging.

**Conclusions:**

This study has demonstrated that neopterin is an excellent biomarker for staging *T. b. gambiense* HAT patients. A rapid diagnostic test for detecting this metabolite in CSF could help in more accurate stage determination.

## Introduction

**Table 1:** Description of the training and the validation cohorts.

	Training cohort (n = 100)		Validation cohort (n = 412)	
	Stage 1 (n = 44)	Stage 2 (n = 56)	Stage 1 (n = 184)	Stage 2 (n = 228)
**Demography**				
Female, n *	19	17	102	98
Age (mean [SD])†	34.4 [±13.4]	31.7 [±12.2]	32.4 [±14.9]	32.8 [±12.7]
**Geographical origin**				
Angola, n	8	13	38	68
Chad, n	8	3	17	13
D.R.C., n	28	40	129	147
**CSF examinations**				
Trypanosome positive, n	0	50	0	166‡
WBC/µL (median [range])	2 [1]–[5]	278.5 [11–1350]	2 [0–5]	161 [1–2000]
**Neurological signs**				
Absent, n	33	3	142	39
Present, n	10	51	36	186
NA, n ||	1	2	6	3

* Fisher’s exact test: training cohort, non significant differences; validation cohort, p value  = 0.0133.

† Mann-Whitney *U* test: training and validation cohort, non significant differences.

‡ Information not available for one patient.

||NA: not available information.

Stage was defined according to WHO guidelines.

Sleeping sickness, or human African trypanosomiasis (HAT), is a parasitic disease that affects rural communities in sub-Saharan Africa [1]. More than 90% of HAT cases are caused by *Trypanosoma brucei gambiense*, responsible for the chronic form of the infection in Western and Central Africa [2]. Sleeping sickness typically progresses from a haemolymphatic first stage (S1) to a meningo-encephalitic second stage (S2), when parasites cross the blood-brain barrier (BBB) and enter the central nervous system (CNS) [3]. As S1 and S2 patients are treated with different drugs, a correct determination of the stage of disease is crucial. *T. b. gambiense* S1 patients are treated with pentamidine, a drug that cannot be used to treat S2 patients due to its low diffusion into the CNS [4]. Most S2 patients were until recently treated with melarsoprol, a toxic arsenic derivate that can cause fatal encephalopathy and for which increased treatment failure rates have been observed in different foci endemic for *T. b. gambiense* HAT [4], [5]. Since 2006 the use of eflornithine has increased and, with the subsequent introduction of nifurtimox-eflornithine combination therapy (NECT), there has been a drastic reduction of the use of melarsoprol in all countries endemic for *T. b. gambiense* HAT [6], [7], [8], [9]. Despite these new advances, difficulties in administration of eflornithine and NECT and associated high costs keep stage determination as a central issue in the management of HAT patients.

**Table 2:** Results obtained for the eight markers assessed on the training cohort.

Marker	[S2]/[S1]	AUC% (95%CI)	Cut-off (95% CI)	SP% (95% CI) *	p value†
**IgM [µg/mL]**	71.9	99.6 (98.9–100)	3.4 (3.3–21)	86.4 (75–95.5)	<0.0001
**Neopterin [nmol/L]**	17.6	99.6 (99–100)	14.3 (13.4–30.3)	84.1 (72.7–93.2)	<0.0001
**MMP-9 [pg/mL]**	38.4	99.4 (98.4–100)	141.2 (126.9–1040)	72.7 (59.1–86.4)	<0.0001
**VCAM-1 [ng/mL]**	3.8	95.5 (91.7–99.4)	15.2 (14.9–21.1)	68.2 (54.6–81.8)	<0.0001
**B2MG [ng/mL]**	5.1	98.4 (96.7–100)	965 (927.5–1577)	63.6 (50–77.3)	<0.0001
**ICAM-1 [ng/mL]**	5.4	97.7 (95.3–100)	1.3 (1.2–2.3)	61.4 (47.7–75)	<0.0001
**CXCL10 [pg/mL]**	32.8	93.3 (88.9–97.8)	757.3 (706.4–1531.7)	43.2 (29.6–59.1)	<0.0001
**CXCL13 [pg/mL]**	641.4	97.6 (94.9–100)	<8.2 (<8.2–64.6)	0 (0–0)	<0.0001

Training cohort (n = 100): Stage 1 n = 44; Stage 2 n = 56. Early-late stage patients were not included.

* Sensitivity was set to 100%.

† Mann-Whitney *U* test.

95%CI = 95% confidence interval; SP%  =  specificity %; SE%  =  sensitivity %.

The reported cut-off and SP% correspond to 100% SE.

Determination of the stage of disease is currently based on examination of the cerebrospinal fluid (CSF) for presence of parasites and counting of white blood cells (WBC). Detection of parasites by microscopy is a specific method and relatively easy to use in the field [10], but limited in sensitivity. To reduce the number of false negatives, i.e. stage 2 patients that would be wrongly treated with ineffective S1 drugs, parasite detection is complemented with counting of white blood cells in the CSF. According to WHO guidelines, S1 is defined as ≤5 WBC/µL and absence of trypanosomes in CSF, while S2 is defined as >5 WBC/µL and/or parasites in CSF [11]. Despite WHO recommendations, a number of countries apply different thresholds to select the appropriate treatment [12], highlighting the lack of a generally accepted gold standard for staging [13]. Furthermore, it has been shown that some patients having between 5 and 20 WBC per microliter of CSF without detected parasites, or patients having ≤5 WBC and presence of parasites (i.e., early-late stage patients) can be cured with S1 drugs [14], [15].

**Table 3:** Results obtained for IgM and neopterin on the validation cohort after application of the cut-off calculated on the training cohort.

Marker	[S2/S1]	AUC% (95% CI)	Applied cut-off	SP% (95% CI)	SE% (95% CI)	p value *
**IgM [µg/mL]**	76.5	96.2 (94.4–98.0)	3.4	86.4 (81–91.3)	91.7 (87.7–95.2)	<0.0001
**Neopterin [nmol/L]**	14.8	95.2 (93.2–97.1)	14.3	87.5 (82.6–91.9)	88.2 (83.8–92.1)	<0.0001

Validation cohort (n = 412): Stage 1 n = 184; Stage 2 n = 228. Early-late stage patients are included in S2 group.

* Mann-Whitney *U* test.

**Table 4:** Results obtained for IgM and neopterin on the validation cohort after application of the cut-off calculated on the training cohort and removal of early-late stage patients (n = 41).

Marker	AUC% (95% CI)	Applied cut-off	SP% (95% CI)	SE% (95% CI)
**IgM [µg/mL]**	99.2 (98.3–100)	3.4	86.4 (81.5–91.3)	98.9 (97.3–100)
**Neopterin [nmol/L]**	99 (98–100)	14.3	87.5 (82.6–91.9)	97.9 (95.7–99.5)

95%CI = 95% confidence interval; SP%  =  specificity %; SE%  =  sensitivity %.

Early-late stage patients (n = 41), i.e. patients having CSF WBC/µL ≤5 and presence of parasites in CSF (n = 4) or patients having 5<WBC/µL≤20 and absence of parasites in CSF (n = 37), were excluded from the group of S2 patients.

**Table 5:** Detailed description of false negative patients obtained according to neopterin and IgM after removal of early–late stage patients from S2 group.

FN Patient	Neopterin<14.3 nmol/L	IgM<3.4 µg/mL	WBC/µL	Parasites	Neurological signs
**# 1**	Yes	Yes	25	Absent	Absent
**# 2**	Yes	No	32	Absent	Absent
**# 3**	Yes	No	261	Present	Present
**# 4**	Yes	Yes	282	Present	Present

FN: false negatives.

During the last decade, many studies have been conducted to rationalise stage determination in sleeping sickness. The use of PCR as surrogate for parasite detection in CSF showed promising results [16], [17], but there is not enough evidence in the literature to define the diagnostic accuracy of PCR in terms of sensitivity and specificity for stage determination [10]. The most promising results were obtained with the detection of intrathecal IgM as indicator of CNS involvement in HAT patients [15], [18], [19]. Detection of IgM was also translated into a rapid field test, LATEX/IgM [18], [20].

**Table 6:** Ability of WBC, IgM and neopterin in discriminating between patients without or with parasites in CSF.

T− vs. T+				
Marker	AUC% (95% CI)	Applied cut-off	SP% (95% CI)	SE% (95% CI)
**WBC (Cells/µL)**	95.1 (92.8–97.4)	5.0	68.6 (62.9–74.3)	98.2 (95.8–100)
**IgM [µg/mL]**	95.2 (93–97.3)	3.4	71.4 (65.7–77.1)	98.2 (95.8–100)
**Neopterin [nmol/L]**	96.0 (93.9–98.0)	14.3	75.1 (69.8–80.4)	97.6 (95.2–99.4)

T−: patients without evidence of parasite in CSF (n = 245); T+: patients with parasite detected in CSF (n = 166). Missing information for 1 S2 patient.

95%CI = 95% confidence interval; SP%  =  specificity %; SE%  =  sensitivity %.

Many other studies that investigated alternative staging markers highlighted a strong correlation between the levels of cytokines, chemokines and other immune-mediators in CSF, and disease progression [21]. In the present study, we assess on a multi-centre cohort the CSF levels of six promising staging markers: B2MG, CXCL10, CXCL13, ICAM-1, VCAM-1 and MMP-9 [22], [23], [24], [25], [26], [27] plus neopterin, an indicator of activation of Th1 immune response [28], already investigated in the staging of *T. b. gambiense* (Krishna S., unpublished) and *T. b. rhodesiense* [29] HAT. The concentration of total IgM was also measured on the same samples, as it currently represents the best alternative to counting WBC. The ability of all these markers to correctly stratify patients classified according to WHO staging criteria was first evaluated on a training cohort comprising 100 *T. b. gambiense* patients. The staging power of the best performing marker, was then validated on a larger multi-centre cohort (n = 412) and compared to IgM and WBC.

**Table 7:** Ability of WBC, IgM and neopterin in discriminating between patients without or with neurological signs.

NS- vs. NS+				
Marker	AUC% (95% CI)	Applied cut-off	SP% (95% CI)	SE% (95% CI)
**WBC (Cells/µL)**	86.2 (82.5–90.0)	5.0	72.4 (65.8–79.0)	83.8 (78.8–88.7)
**IgM [µg/mL]**	84.5 (80.6–88.5)	3.4	71.8 (65.2–78.5)	80.6 (75.2–85.6)
**Neopterin [nmol/L]**	88.2 (84.9–91.4)	14.3	78.5 (72.4–84)	81.5 (76.6–86.5)

NS−: patients without neurological signs (n = 181); NS+: patients with neurological signs (n = 222). Missing information for 9 patients.

95%CI = 95% confidence interval; SP%  =  specificity %; SE%  =  sensitivity %.

## Methods

### Ethics Statement

The National Ethical Committees of the countries where samples were collected (Angola, Chad and the Democratic Republic of the Congo – D.R.C.) approved the studies. HAT patients gave written informed consent before enrolment. Children (<18 years) or patients with altered mental status, a common condition in late stage HAT, were included only after written informed consent from a parent or a guardian. All patients had the option of withdrawing from the studies at any time.

**Figure 1:**
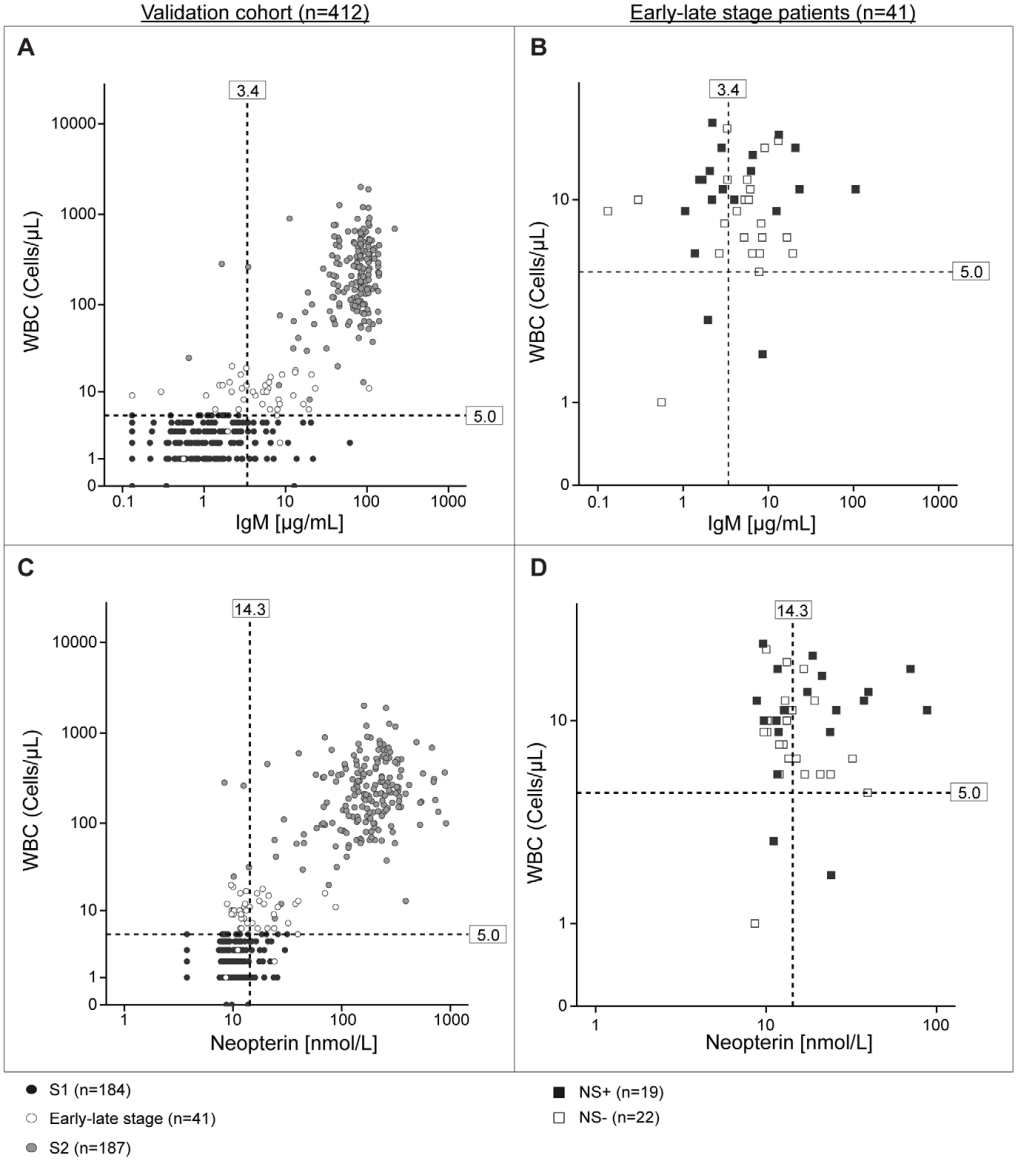
Classification of HAT patients according to WBC and IgM, or according to WBC and neopterin. A) Comparison of the classification of HAT patients (validation cohort) according to WBC and IgM. The staging cut-off of 5 WBC/µL recommended by WHO is reported on the graph as well as the staging cut-off of 3.4 µg/mL for IgM calculated on the training cohort. Black dots represent stage 1 patients, white dots represent early-late stage patients and gray dots represent stage 2 patients. B) Detailed classification of early-late stage patients (n = 41) according to IgM. Colours indicate the absence (white) or presence of neurological (black) signs. The staging cut-off of 5 WBC/µL recommended by WHO is reported on the graph as well as the staging cut-off of 3.4 µg/mL for IgM calculated on the training cohort. C) Comparison of the classification of HAT patients (validation cohort) according to WBC and neopterin. The staging cut-off of 5 WBC/µL recommended by WHO is reported on the graph as well as the staging cut-off of 14.3 nmol/L for neopterin calculated on the training cohort. Black dots represent stage 1 patients, white dots represent early-late stage patients and gray dots represent stage 2 patients. D) Detailed classification of early-late stage patients (n = 41) according to neopterin. Colours indicate the absence (white) or presence (black) of neurological signs. The staging cut-off of 5 WBC/µL recommended by WHO is reported on the graph as well as the staging cut-off of 14.3 nmol/L for neopterin calculated on the training cohort. NS+: presence of neurological signs; NS-: absence of neurological signs.

### Patients

Cerebrospinal fluid samples of 1028 patients with evidence of trypanosomes in blood, lymph or CSF were received at the University of Geneva. The samples originated from patients identified through active and passive case finding between 2005 and 2009 in Angola, Chad and the Democratic Republic of the Congo. Sample collection was done in the context of prospective diagnostic studies (THARSAT [30], NEUROTRYP [22], [23] and FIND/CD19), or was directed by the WHO for a HAT specimen bank (Table S1 and Figure S1).

Stage determination and treatment were performed in the designated treatment centres or by medical teams at the site of active screening, according to the policies of the national sleeping sickness control programs. The number of WBCs in CSF was determined by microscopy, and presence of parasites was determined during WBC counting or by using the modified single centrifugation method [31]. Samples were aliquoted and stored at −80°C or in liquid nitrogen. The levels of biomarkers were evaluated on the CSF samples between December 2009 and August 2010.

For the present study, all patients were classified as S1 or S2 according to WHO guidelines, i.e. S1 when ≤5 WBC/µL and no parasites in CSF, S2 when >5 WBC/µL and/or trypanosomes in CSF, regardless of the cut-offs applied at the country level. Exclusion criteria were: age <12 years, spontaneous withdrawal from the study, insufficient volume of CSF, haemorrhagic CSF (>100 red cells/µL), or missing information to classify patients as S1 or S2 according to WHO criteria. Information on the neurological status of patients, including daytime somnolence, sensory and gait disturbances, presence of primitive reflexes (palmomental and perioral reflexes), modified tendon reflexes (exaggeration or abolition), Babinski’s sign, abnormal movements such as tremors (fine, diffused and generalized), was recorded when available. Due to the absence of systematic screening for malaria or HIV co-infections, we considered their prevalence not different between S1 and S2 patients.

Five hundred and twelve patients were considered eligible for the present study and were used to create two study cohorts. A first training cohort of 100 patients was established with 44 S1 and 56 S2 randomly chosen from among all eligible patients. For the selection of S2 patients, those considered to be early-late stage (i.e., patients having 5<WBC/µL ≤20 and absence of parasites, or patients having ≤5 WBC/µL and presence of parasites) [14], [15] were not included. The remaining 412 patients were included in the second cohort (i.e. the validation cohort). Estimating a staging AUC ≥90% for each marker based on the results obtained on the training cohort, the calculated power of the test on the validation cohort was equal to 1.

### Immunoassay Procedures

Commercially available human ELISA kits were used to determine CSF levels of CXCL13 (R&D Systems, UK), IgM (ICL, OR, USA), B2MG (Calbiotech, CA, USA) and neopterin (BRAHMS, Germany). Multiplex bead suspension assays (mBSA) were used to measure CXCL10 (Bio-Rad, CA, USA), MMP-9, VCAM-1 and ICAM-1 (R&D Systems). The assays were performed following manufacturers’ instructions. For each assay the limit of detection (LOD) was calculated as the mean concentration of the lowest standard less 2 standard deviations. To all outliers (≤LOD), a value corresponding to the mean of LODs divided by 2 was assigned. Variability (CV) between assays, evaluated using internal quality controls, was below 25%.

### Statistical Analysis

Data analysis was performed using IBM SPSS Statistics version 20.0.0 (IBM, NY, USA), GraphPad PRISM version 4.03 (GraphPad Software Inc.) and S+ version 8.1 (TIBCO, Software Inc.). All statistical tests were two-tailed. Differences between two groups were assessed with the Mann-Whitney *U* test. For each biomarker, receiver-operating characteristic (ROC) curves, area under the curve (AUC), specificity (SP) and sensitivity (SE) were calculated from the pROC package [32]. To minimize the number of false negatives, the staging cut-offs on the training cohort were calculated as the threshold corresponding to 100% sensitivity.

On the validation cohort, the specificity and sensitivity for discrimination between early and late stage patients were obtained by applying the cut-offs calculated on the training cohort. The same analysis was done on patients classified according to the absence (T−) or presence (T+) of parasites in the CSF; or according to the absence (NS−) or presence (NS+) of neurological signs. Confidence intervals at the 95% level on AUC, sensitivity, specificity and cut-off were computed with pROC.

This work conforms to the STARD guidelines for reporting of studies on diagnostic accuracy.

## Results

### Training Cohort

When assessed on a the training cohort, comprising 44 early stage and 56 late stage *T. b. gambiense* patients (Table 1), the seven markers (CXCL10, CXCL13, neopterin, B2MG, ICAM-1, VCAM-1 and MMP-9) showed high ability to discriminate between the two groups of patients (AUC >90% and p value <0.0001, Mann-Whitney *U* test) (Table 2). The same results were obtained with IgM, confirming its value as a staging marker [18]. To reduce the number of false negatives, i.e. S2 patients wrongly classified as S1, the threshold corresponding to 100% sensitivity was calculated for each marker. Only IgM and neopterin were >80% specific at 100% sensitivity. In particular, with a threshold concentration of 14.3 nmol/L (13.4–30.3 95% CI), neopterin was 84% specific. Similar results were obtained with IgM (86.4% specificity) at a threshold concentration of 3.4 µg/mL (3.3–21 95% CI) (Table 2).

### Validation Cohort

Neopterin and IgM were then measured on a validation cohort comprising 184 S1 and 228 S2 *T. b. gambiense* patients (Table 1). CSF neopterin at a threshold concentration of 14.3 nmol/L discriminated between early and late stage patients with 87.5% specificity (82.6–91.9 95% CI) and 88.2% sensitivity (83.8–92.1 95% CI) (Table 3). This means that 23 out of 184 S1 patients were classified as stage 2 by neopterin (false positives) and 27 out of 228 S2 were classified as S1 (false negatives). Among these 27 false negatives, twenty three belonged to the early-late stage group, i.e. patients having between 5 and 20 WBC/µL with no trypanosomes in CSF or ≤5 WBC/µL and presence of trypanosomes in CSF. When all early-late stage patients (n = 41) were excluded from the S2 group, the number of false negatives was only 4 patients, thus increasing the sensitivity of neopterin in determining the disease stage to 97.9% (95.7–99.5 95% CI), (Table 4). Two of these 4 patients had ≤32 WBC/µL and neither showed evidence of parasites in CSF nor presence of neurological signs (Table 5).

With a staging cut-off of 3.4 µg/mL, IgM correctly discriminated S1 and S2 patients, classified according to WHO guidelines, with 86.4% (81–91.3 95% CI) specificity (25/184 false positives) and 91.7% (87.7–95.2 95% CI) sensitivity (19/228 false negatives) (Table 3). When early-late stage patients were excluded, the number of false negatives decreased to 2 patients thus increasing the sensitivity of IgM in disease staging to 98.9% (97.3–100 95%CI) (Table 4, Table 5).

To compare neopterin and IgM with WBC, patients were classified according to absence or presence of trypanosomes in CSF or according to absence or presence of neurological signs, as these two conditions can be indicative of an advanced stage of disease. The ability of the two markers and WBC to discriminate between the two groups was then assessed through ROC curves. Neopterin (14.3 nmol/L) was the best discriminator between patients without parasites in CSF (n = 245) and those with detected parasites (n = 166), showing higher specificity (75.1%, 69.8–80.4 95% CI) than WBC (68.6%, 62.9–74.3 95% CI) for similar levels of sensitivity (Table 6).

When patients were grouped based on the absence (n = 181) or presence (n = 222) of neurological signs, the specificity and sensitivity of both neopterin and IgM were comparable to WBC (Table 7).

Finally we assessed how neopterin and IgM classified early-late stage patients (n = 41), defined as S2 according to WHO guidelines. Based on the two markers, respectively 23 (56%) and 17 (41%) of these patients were classified as S1 instead of S2. Most of the early-late stage patients (71%) were classified as stage 1 by both neopterin and IgM. However, no relationship between the presence of neurological signs and the levels of the two markers could be demonstrated in the analyzed patients (Figure 1).

## Discussion

The lack of a generally accepted gold standard highlights the need of new tools that could replace WBC counting and complement trypanosome detection in the staging of *T. b. gambiense* sleeping sickness patients [13], [33]. The aim of the present study was to compare on a large multi-centre cohort of *T. b. gambiense* patients the ability of the best staging markers in CSF proposed in the literature [13], [21], [33] to discriminate between S1 and S2 HAT patients. This is the first time that neopterin, IgM, CXCL13, ICAM-1, VCAM-1, MMP-9, CXCL10 and B2MG have been assessed on the same training population encompassing 100 *T. b. gambiense* HAT patients. This evaluation allowed a better comparison of their individual performances, leading to the selection of neopterin as a promising new staging marker for *T. b. gambiense* sleeping sickness.

Due to the risk of relapses and even death when S2 patients are misdiagnosed and treated as S1 patients, a highly sensitive marker is needed in order to reduce the number of false negative patients. A cut-off corresponding to 100% sensitivity was thus established on the training cohort, and neopterin emerged as the most powerful staging marker with specificity close to that of IgM, the best alternative to WBC proposed so far.

Neopterin and IgM were further tested on a validation cohort encompassing 412 *T. b. gambiense* patients and the cut-offs calculated on the training cohort applied. The results obtained confirmed previously published data on the high staging power of IgM, for which a rapid, field applicable agglutination test was developed [18], as well as preliminary data on the high correlation between neopterin and WBC counts (S. Krishna, unpublished). The decreased sensitivity observed on the validation cohort was due to the presence of early-late stage patients in the S2 group. These patients are the most critical because in some cases they have been reported to be cured with pentamidine [14], [34]. When these patients were omitted from the validation cohort, the sensitivity of neopterin increased to 97.9% meaning that only 4 S2 patients, including 2 with trypanosomes in the CSF, were classified as false negatives. The value of neopterin as a staging tool was also supported by the results obtained for IgM, which showed very similar performance. Furthermore, both IgM and neopterin were shown to correlate with presence of parasites in CSF and neurological signs with the same accuracy as WBC count.

A rapid assay for detection of neopterin in CSF might have potential to replace WBC counting for diagnosis of the meningo-encephalitic stage of HAT, in combination with parasite detection in CSF. These important perspectives in the field of staging do not find application in the diagnosis of the disease, which is based on detection of parasite-specific antibodies using the CATT test as a screening tool, followed by parasitological confirmation of disease. It might also show potential for assessment of cure after treatment, within a shorter period compared to IgM and WBC count [30]. Neopterin is a stable catabolic product of guanosine triphosphate (GTP) that is produced by interferon (IFN)-γ activated macrophages and dendritic cells [35]. It has been proposed as a marker of disease activity in tuberculosis [36], [37] and of CNS involvement in HIV and *T. b. rhodesiense* HAT [29]. A simple dipstick assay for semi-quantitative detection of neopterin in serum has already been found useful and practicable for other infectious diseases [38], indicating the potential to assess neopterin in CSF with a rapid diagnostic test.

The present study had a number of limitations that need to be considered. An important weakness was the absence of highly detailed and standardised information on patients’ neurological status or presence of concomitant infections such as HIV and malaria, which could help in interpretation of data. The markers reported here for staging are not specific for HAT. Sleeping sickness patients are exposed to other infections, and neopterin has already been proposed as marker for other diseases that are endemic in Africa [36]. Thus, the effect of concomitant diseases on the levels of the markers should be investigated further.

Another drawback is the absence of a gold standard to which the performance of neopterin, as well as the other molecules analysed, could be compared. In an effort to overcome this deficiency, the staging performance was correlated to the presence of parasites in CSF, neurological abnormalities and WBC counts, parameters used for disease staging in practice. Furthermore, neopterin was also compared to IgM, already validated as a staging marker [15], [18]. However, within the study cohorts, the methods used for parasite detection, WBC count and assessing neurological signs were not standardised, thus reducing the accuracy of original staging. Finally, the utility of the tested marker, including neopterin, in correct staging of early-late stage patients, i.e. patients having between 5 and 20 WBC/µL and no parasites in CSF, or patients having ≤ 5 WBC/µL and trypanosomes in CSF, cannot be assessed on the current cohort. Some early-late stage patients have previously been reported to be cured with pentamidine [34]. In the studied cohort, some of the early-late stage patients could have benefited from treatment with S1 drugs, as suggested by levels of neopterin and IgM that were below the proposed threshold values.

The markers evaluated in the current study still rely on examination of CSF, which is the basis of actual staging. Ideally, alternative markers for staging should be detectable in plasma, thus avoiding lumbar punctures. Unfortunately, none of the markers investigated here was able to distinguish between S1 and S2 patients when measured in plasma (data not shown).

In conclusion this study highlights the value of neopterin in CSF as a marker for the identification the meningo-encephalitic stage of *gambiense* HAT. A rapid diagnostic test for detection of this metabolite in patients’ CSF could become a valuable alternative to counting of WBC, still to be combined with parasite detection in CSF. The value of neopterin for follow-up after treatment should be investigated as well as its ability to stage *Trypanosoma brucei rhodesiense* HAT.

## Supporting Information

Figure S1 **Flow chart representing the collected CSF samples.**
(TIF)

Table S1 **Description of the prospective diagnostic studies from which patients were obtained.**
(DOCX)
