# Supplementary figures and images for: Cerebrospinal Fluid Neopterin as Marker of the Meningo-Encephalitic Stage of Trypanosoma brucei gambiense Sleeping Sickness
Source: PLoS One. 2012 Jul 18;7(7):e40909. doi: 10.1371/journal.pone.0040909 (PMC3399808; doi:10.1371/journal.pone.0040909)

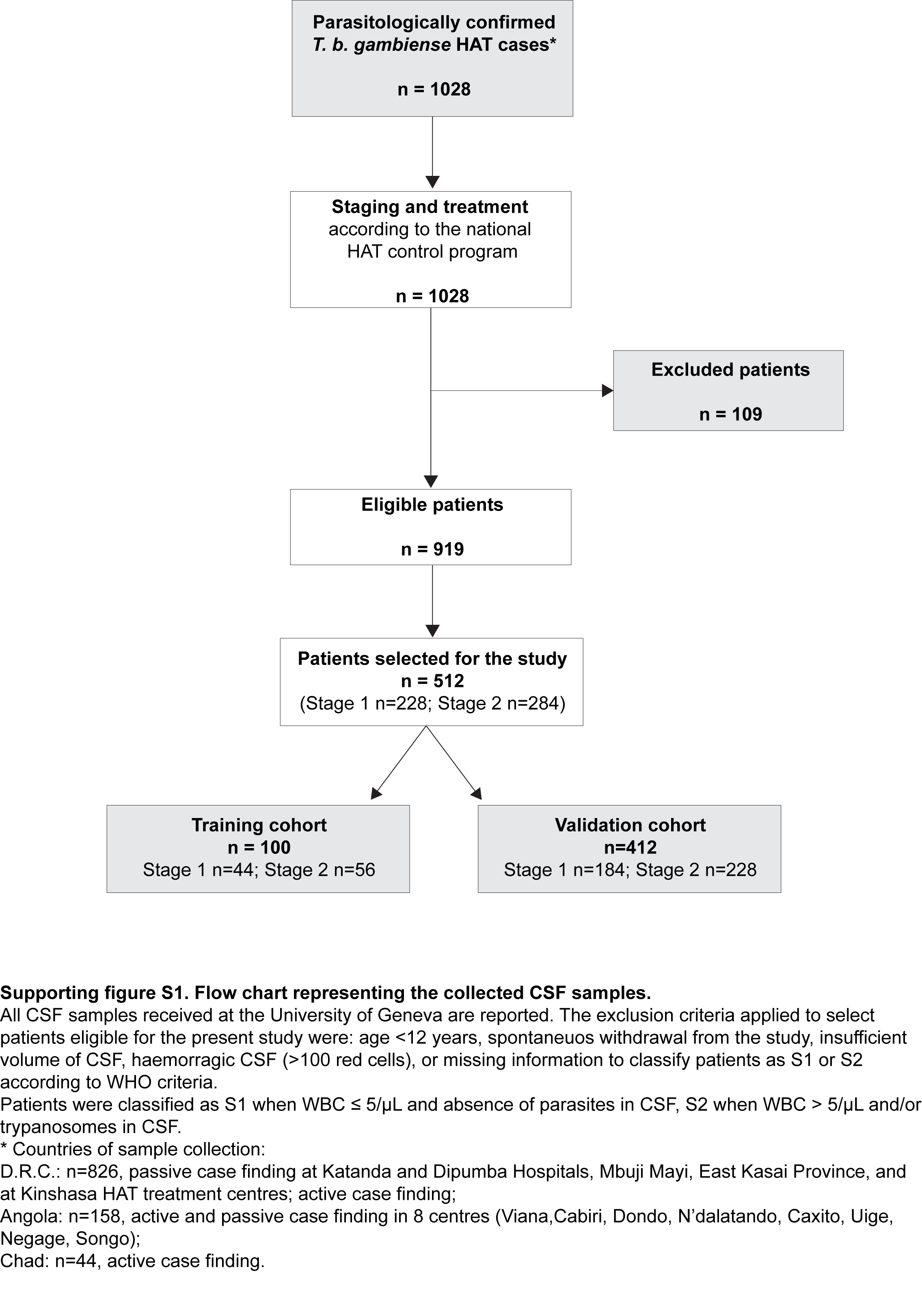

Supplement: Figure S1 — Flow chart representing the collected CSF samples. (TIF) [file pone.0040909.s001.tif]
