# Supplementary material for: Cerebrospinal Fluid Neopterin as Marker of the Meningo-Encephalitic Stage of Trypanosoma brucei gambiense Sleeping Sickness
Source: PLoS One. 2012 Jul 18;7(7):e40909. doi: 10.1371/journal.pone.0040909 (PMC3399808; doi:10.1371/journal.pone.0040909)
Supplement: Table S1 — Description of the prospective diagnostic studies from which patients were obtained. (DOCX) [file pone.0040909.s002.docx]

**Supporting Table S1. Description of the prospective diagnostic studies from which patients were obtained.**

| **Study Name** | **THARSAT** | **NEUROTRYP** | **WHO HAT Specimen bank** | **FIND/CD19** |
| --- | --- | --- | --- | --- |
| **Country and place of sample collection** | **DRC**: Dipumba Hospital, Mbuji Mayi, East Kasai Province | **DRC**: Dipumba Hospital, Mbuji Mayi, East Kasai Province | **DRC**: Hôpital du Roi Baudouin and Maluku HAT treatment centre, Kinshasa; Miabi and Tshilenge Mobile Units, Kasai Oriental; Katanda and Dipumba HAT treatment centres, East Kasai  **CHAD**: Mobile Unit in Mandoul | **ANGOLA**: Centres of Viana, Cabiri, Dondo, N’dalatando, Caxito, Uige, Negage, and Songo |
| **Time period and method of sample collection** | May 2005 – February 2006  Active and passive case finding | March 2007 – November 2008  Active and passive case finding | August 2006 – March 2009  Active and passive case finding | May 2008 – May 2009  Active and passive case finding |
| **Inclusion Criteria** | Presence of trypanosomes in lymph, blood or CSF  Age ≥ 12 years  Patients living in a 100 km perimeter around the treatment centre  Written informed consent | Presence of trypanosomes in lymph, blood or CSF  Age ≥ 12 years  Patients living in a 100 km perimeter around the treatment centre  Written informed consent | Presence of trypanosomes in lymph, blood or CSF  Age ≥ 12 years  Written informed consent  Adequate quantity of specimen | Presence of trypanosomes in lymph, blood or CSF  Age ≥ 10 years  Written informed consent |
| **Exclusion Criteria** | Age < 12 years  Moribund conditions  Concurrent serious illness (tuberculosis, bacterial or criptococcal menigitis) Pregnancy  No guarantee for follow-up  Heamorrhagic CSF | Age < 12 years  Moribund conditions  No guarantee for follow-up  Heamorrhagic CSF | Age < 12 years  Refusal of provide informed consent  Inability to get adequate quantity of sample  Heamorrhagic CSF (≥ 100 red cells/µl) | Age < 10 years  Moribund conditions  Concurrent serious illness Pregnancy  Heamorrhagic CSF (≥ 500 red cells/µl)  Refusal of participation to the study at any moment |
| **Method for trypanosome finding in CSF** | Modified single centrifugation | Modified single centrifugation | According to national control program | Modified single centrifugation |
